# Variational problems in the theory of hydroelastic waves

**DOI:** 10.1098/rsta.2017.0343

**Published:** 2018-08-20

**Authors:** P. I. Plotnikov, J. F. Toland

**Affiliations:** 1 Voronezh University, Universitetskaya pl., Voronezh 394018, Russia; 2 Lavrentyev Institute of Hydrodynamics RAS, Lavrentyev pr. 15, Novosibirsk 630090, Russia; 3 Department of Mathematical Sciences, University of Bath, Bath BA2 7AY, UK

**Keywords:** hydroelastic waves, Willmore functional, theory of shells

## Abstract

This paper outlines a mathematical approach to steady periodic waves which propagate with constant velocity and without change of form on the surface of a three-dimensional expanse of fluid which is at rest at infinite depth and moving irrotationally under gravity, bounded above by a frictionless elastic sheet. The elastic sheet is supposed to have gravitational potential energy, bending energy proportional to the square integral of its mean curvature (its Willmore functional), and stretching energy determined by the position of its particles relative to a reference configuration. The equations and boundary conditions governing the wave shape are derived by formulating the problem, in the language of geometry of surfaces, as one for critical points of a natural Lagrangian, and a proof of the existence of solutions is sketched.

This article is part of the theme issue ‘Modelling of sea-ice phenomena’.

## 1. Nonlinear hydroelastic waves

We consider the motion of an ideal incompressible liquid under an elastic sheet and develop a framework in which to study the existence and properties of periodic hydroelastic travelling waves that are stationary with respect to a frame moving with the wave, thereby possibly describing the propagation of periodic waves of finite amplitude on the surface of an ocean covered by ice. There are many approaches to linear hydroelastic waves but here we concentrate on nonlinear theories. One of the first rigorous result in this direction was due to Toland [1], who showed the existence of two-dimensional hydroelastic travelling waves of finite amplitude under an assumption that the mechanics of the elastic sheet is modelled by Cosserat theory and its density is zero. That work was extended to the case of non-zero density by Plotnikov & Toland [2], who later considered the modelling of non-stationary wave problems [3]. For various models of nonlinear two-dimensional hydroelastic waves, local existence and uniqueness results for the Cauchy problem were obtained by Ambrose & Siegel [4] and Liu & Ambrose [5].

In a particular nonlinear two-dimensional theory, the elastic sheet is considered to be an Euler elastica with stretching energy density proportional to the length element of the sheet. In this setting, travelling two-dimensional hydroelastic waves were investigated by Guenne & Pǎrǎu [6], Vanden Broeck & Pǎrǎu [7,8], Deacon *et al.* [9], Milewski *et al.* [10] and Wang *et al.* [11]. In related work, numerical experiments by Milewski *et al.* [12] and Gao *et al.* [13] exhibit a wide variety of different hydroelastic wave types. Three-dimensional waves were studied by Milewski & Wang [14] and by Groves *et al.* [15], who developed a variational approach to the problem.

Here we focus on a three-dimensional model where the underlying equations have Hamiltonian structure and the travelling hydroelastic waves problem can be reduced to the existence of critical points of a Lagrangian which involves the full hydroelastic energy and an inertial term. The resulting variational problem, which involves joint minimization of the Willmore functional and a Dirichlet integral, is similar to one from conformal geometry.

We assume that the flow occupies a domain 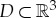 of points *x* = (*x*^1^, *x*^2^, *x*^3^) bounded by the elastic sheet *S*: = ∂*D* which is itself contained within a horizontal layer and that *D* contains a half space. It is not assumed that *S* is the graph of a function. More precisely,

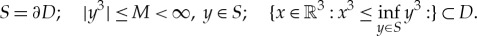


Considering only periodic waves we assume that *D* is periodic:

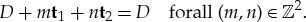
Here the linearly independent vectors ***t***_*i*_ = (*t*^1^_*i*_, *t*^2^_*i*_, 0),  *i* = 1, 2, form a lattice of periods in the space 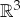 and we denote the fundamental cell by
1.1
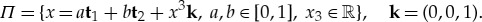
As in elasticity theory, we assume the elastic shell on the surface can be parametrized as follows:

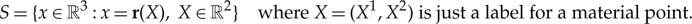
It is natural to assume that in the reference frame the shell has periodic structure with periods ***l***_*i*_ = (*l*^1^_*i*_, *l*^2^_*i*_, 0), *i* = 1, 2, with fundamental cell
1.2
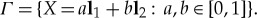
Since it is not assumed that *S* is the graph of a function, this means that ***r*** admits the representation
1.3
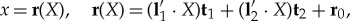
where (***l***′_1_, ***l***′_2_) is the dual lattice defined by the relations ***l***_*i*_′ · ***l***_*j*_ = *δ*_*ij*_ and 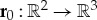 is a ***l***-periodic mapping. While the physical periods ***t***_*i*_ are prescribed, the periods ***l***_*i*_ of the reference frame are unknown and form part of a solution to the hydroelastic wave problem.

### (a) Travelling waves, moving surfaces

Our goal is to study hydroelastic travelling waves which propagate on the surface with constant speed. A peculiarity of this problem is that there are two speeds of wave propagation. To see this assume that a material surface *S* at rest has parametrization *x* = ***r***(*X*). If it is moving with constant speed, the moving surface *S*_*t*_ admits the parametrization *S*_*t*_:*x* = ***r***(*X* − *t****c***) + ***V***, 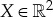, where ***V*** is the velocity of the surface *S*_*t*_ moving as rigid body in 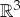, and ***c*** = (*c*_1_, *c*_2_) gives the velocity of motion of material points relative to *S*_*t*_. For water waves this observation is not important because only the moving shape of the water surface is visible and the motion of the liquid particles on the surface is not. Moreover, the velocities and accelerations of particles on the surface are determined by the equations of hydrodynamics, not by the geometry of the free surface. However, for hydroelastic waves, the equations of hydrodynamics do not control the distribution of elastic material on the surface.

In the equation for a linear elastic beam
1.4
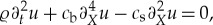
the fourth-order term corresponds to bending and the second-order term to stretching. In the case of travelling waves, which propagate in the beam with velocity *c*, equation (1.4) becomes an ordinary differential equation

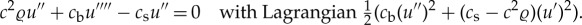
This simple example shows that *c*, which is the speed of elastic wave propagation, appears if the elastic material has a non-zero density. Moreover, its effect in the energy balance is of the same order as that of stretching.

### (b) The equations

After the change of variables *x* → *x* − *t****V*** and *X* → *X* − *t****c***, the time variable can be eliminated and the hydroelastic travelling wave problem reduced to a stationary-free boundary-value problem in which the domain *D* is unknown. To this end, assume that the flow is irrotational, periodic, satisfies the kinematic condition at the free surface and tends to uniform flow with speed ***V*** at infinity. After rotation and scaling, we can suppose that ***V*** = ***i***: = (1, 0, 0). Therefore, the fluid velocity 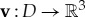 admits the representation ***v*** = ∇*φ*, *φ*(*x*) = *x*^1^ + *Φ*(*x*) where, from the above assumptions, the potential *Φ* satisfies the following equations and boundary conditions:
1.5
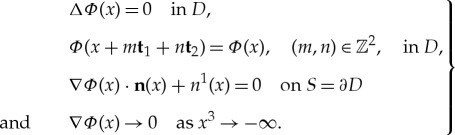
Here ***n*** = (*n*^1^, *n*^2^, *n*^3^) is the unit outward normal on *S* = ∂*D*. Recall that the fluid pressure *p* for steady irrotational flow is given by the Bernoulli equation

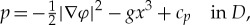
where *c*_*p*_ is a constant.

## 2. Lagrangian

In the absence of viscous dissipation of energy, this hydroelastic wave problem can be formulated variationally as one for critical points of the action functional 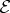, which is the difference between the full energy of the hydroelastic system and the work of the inertial forces. The full energy comprises the kinetic and gravitational potential energies of the fluid and the elastic energy of the sheet, and traditionally the elastic energy density of the sheet is decomposed as the sum the bending and stretching energies. The justification for this splitting is that these parts correspond to terms of different order, as functions of a small parameter which characterizes the thickness of the plate, in an asymptotic expansion of the elastic plate energy in three dimensions. Therefore, in what follows we take the action functional in the form
2.1
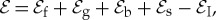
where 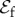 and 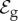 are renormalized kinetic and gravitational potential energies of the fluid per period, 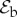 and 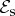 are the bending and stretching energies of the elastic surface *S*, and 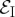 is the work of the inertial forces per period. To represent these quantities as integrals, let *Ω* = *D*∩*Π* and *S*_*Π*_ = *S*∩*Π*, respectively, the intersections of the flow domain and free boundary with the fundamental cell *Π* is given by (1.1). In general, the surface *S*_*Π*_ consists of a countable set of disjoint connected components and it is convenient to replace it with the connected surface *Σ*: = ***r***(*Γ*), where the reference fundamental cell *Γ* of periods is given by (1.2), and ***r*** is the parametrization of *S* satisfying condition (1.3). It follows from the periodicity conditions that the identity

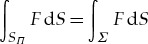
holds for every function *F* which is locally integrable over *S*.

### (a) Kinetic and gravitational potential energies of fluid

The renormalized kinetic and gravitational potential energies are
2.2
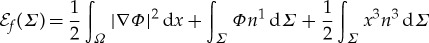
and
2.3
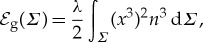
where *Φ* is a solution to Neumann problem (1.5) and λ^−2^ is the Froude number. Note that the potential *Φ* is a solution of a variational problem for the Dirichlet integral. Denote by *H*^1,2^_♯_(*D*), the space of all functions 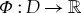 satisfying the periodicity condition

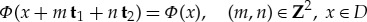
and having the finite semi-norm

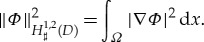
After identifying functions which differ one from another by a constant it becomes the Hilbert space 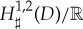. By virtue of Dirichlet's principle, we have
2.4
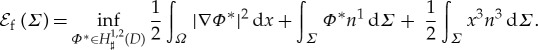
This means that 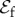 and 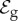 are completely determined by the surface *Σ*. Moreover, if the wave is bounded, i.e. |*y*^3^| < *M* for *y*∈*S*, then the surface integrals in (2.2)–(2.3) can be transformed into volume integrals by integration by parts:

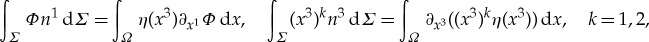
where *η* is an arbitrary smooth function of *x*^3^ which is 1 for *x*_3_ > − *M* and 0 for *x*^3^ < − 2*M*. Therefore, the kinetic and gravitational potential energies are well defined for all continuous surfaces *S*, without self-intersections, which are bounded in the *x*^3^-direction.

### (b) Elastic energy: preliminaries

To derive the integral representations for bending and stretching energies, we recall some basic facts from differential geometry. Henceforth, we adopt the notation ∂_*i*_: = ∂/∂*X*^*i*^, *i* = 1, 2, for partial derivatives with respect to the reference variables *X*_1_, *X*_2_. For a rectifiable surface *S* given by *x* = ***r***(*X*), the normal vector at ***r***(*X*) is

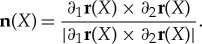
The first fundamental form of *S* defines the metric ***g*** on *S* induced by the metric of the ambient space 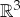, which means that the length d*s* of the element (d*X*^1^, d*X*^2^) is given by
2.5
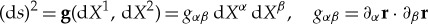
and the area element d*S* of the surface *S* is given by
2.6
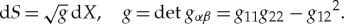
The second fundamental form ***A*** of *S* is defined by
2.7
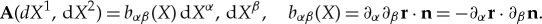
The mean curvature ***H***, the Gauss curvature ***K*** and the length |***A***| of the second fundamental form are algebraic invariants of the symmetric matrix ***g***^−1/2^***Ag***^−1/2^,
2.8
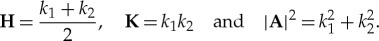
Here the principle curvatures *k*_*i*_ are the eigenvalues of ***g***^−1/2^***Ag***^−1/2^. By virtue of the Gauss–Bonnet theorem, the integral of the Gauss curvature over *Σ* = ***r***(*Γ*) is a topological invariant. If the parametrization of *S* satisfies the periodicity condition (1.3), then *Σ* may be considered as a two-dimensional topological torus with genus 1. Then from the Gauss–Bonnet theorem, it follows that
2.9
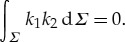
Conformal (or isothermal) coordinates on the elastic shell play an important role in the analysis of stretching energy. Recall that the parametrization *x* = ***r***(*X*) of the surface *S* is conformal if *g*_11_ = *g*_22_ = *e*^*f*^, say, and *g*_12_ = 0, in which case
2.10
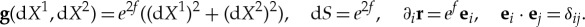
The triplet (***e***_1_, ***e***_2_, ***n***) defines an orthonormal moving frame on *S*. In its turn, the orthogonal unit vectors ***e***_1_ and ***e***_2_ form a moving Coulomb frame [16, p. 167] on *S*. They are connected with the conformal factor *e*^2*f*^ by the relation, see [16, (5.11)],

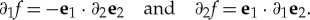


### (c) Bending energy

In their classic textbook Landau & Lifschitz [17] considered the bending energy density of an elastic sheet as a product of the area element with a linear combination of the squared mean curvature (the Willmore energy density) and the total Gauss curvature. Recall that the product of the Gauss curvature and the area element is a Jacobian, the integral of which over a closed topological manifold is a topological invariant (a so-called null Lagrangian), see identity (2.9) for a torus. An explicit expression for the bending energy for hyperelastic materials based on rigorous asymptotic analysis was derived in [18] where it was shown that the bending energy density of a elastic shell can be represented as

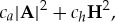
where |***A***| and ***H*** are the length, respectively, of the second fundamental form ***A*** and the mean curvature ***H*** of *S*, and the positive constants *c*_*a*_ and *c*_*h*_ are determined by the stored energy function of the particular hyperelastic material. It follows from this and identities (2.8)–(2.9) that we can take the bending energy 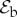 in the form
2.11
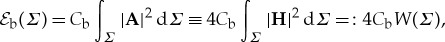
where the constant *C*_b_ is determined by the material and *W*(*Σ*) is the Willmore functional of *Σ*.

The Willmore functional was first proposed as a model for bending elastic energy by Poisson [19] in 1816 and again by Kirchhoff [20] in 1850. More recently, it has found applications in cell biology. For example, *W*(*Σ*) is the principle part of the Helfrich functional [21] in the modelling of bilayer lipid membranes in cell mechanics. The first mathematical treatment of the Willmore functional is due to Blaschke and Thomsen [22], who proved that the integral of the squared mean curvature over a closed surface *M* is a conformal invariant. This means that it is invariant with respect to translations, rotations and dilatations of *M*. It is also invariant with respect to the inversion *x* → |*x*|^−2^*x* provided that the pole of inversion does not belong to *M*. In particular, the Willmore energy equals 4*π* for every spherical surface. In fact, the Willmore energy is the only conformal invariant up to a null Lagrangian. Blaschke and Thomsen also derived the formula for the variation of the mean square curvature functional. These results were rediscovered by Willmore [23], who calculated the value of the Willmore energy for tori and considered the variational problems related to this functional. We refer to the papers by Bernard& Rivière [24], Kuwert & Schatzle [25], Simon [26] and the monograph by Hélein [16] for the references and the state of art in this domain.

### (d) Stretching energy and the inertial term

For hyperelastic materials, when the stretching energy 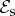 corresponds to the first term in an asymptotic expansion of three-dimensional elastic plate energy as a function of the (small) thickness of the plate, a rigorous derivation of an integral representation for the stretching energy was given in [27,28]. There it was shown that, in contrast to the case of bending energy, stretching energy is very sensitive to the structure of the three-dimensional energy density of a particular hyperelastic material. In classical linear elasticity theory, the stretching energy has the simplified form:

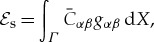
where the constants 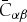 are the entries of a positive symmetric matrix 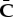 and *Γ* is the fundamental cell of periods in the reference frame defined by (1.2). Note that 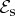 depends on the choice of the parametrization *x* = ***r***(*X*) of the surface *S*, i.e. 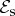 is not a geometric invariant.

In terms of ***c***, the velocity of elastic wave propagation, the work 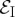 of the inertial forces is given by

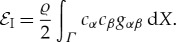
Here ϱ is the density of the elastic sheet *S* and *c*_*α*_,  *α* = 1, 2, are the components of the vector ***c***. It follows that the difference 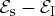 is linear in the entries *g*_*αβ*_, and quadratic in the tangential vectors ∂_*α*_***r***. Since the form 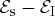 depends on the wave speed ***c***, it may be positive, non-negative or indefinite, corresponding, respectively, to subsonic, sonic or supersonic stretching waves. In this paper, we restrict consideration to subsonic waves in which case 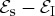 in (2.1) has the form
2.12
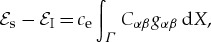
where the constant *c*_e_ is chosen so that the symmetric matrix ***C*** = (*C*_*αβ*_) satisfies
2.13
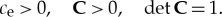


### (e) Specifications of the periodic lattice (***l***_1_, ***l***_2_)

We now specify the lattice of periods in the reference frame using the symmetry of the quadratic form (2.12). First, note that this form is invariant with respect to dilatations of the reference frame. Next, for a linear change of the variables *X* = ***U****X*′ with a constant matrix ***U***,

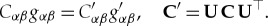
Hence the energy density of 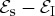 is invariant under all linear transforms of the reference variables with matrices ***U*** satisfying the conditions ***U*** ***C*** ***U*** → *p* = ***C*** (from which it follows that det ***U*** = 1). Since ***C*** is symmetric, the class of admissible matrices ***U*** includes all matrices of the form ***U*** = ***C***^1/2^***OC***^−1/2^, where ***O*** is an arbitrary unitary matrix, ***OO*** → *p* = ***I***. Thus, with ***i*** = (1, 0) and ***j*** = (0, 1), we can choose an appropriate matrix ***O*** such that ***C***^1/2^***OC***^−1/2^***i*** = const. ***l***_1_. Then after scaling we can choose *X*′ so that in new reference variables the area of the fundamental cell *Γ*′ is 1. Hence, without loss of generality we can assume that the periods ***l***_*i*_ have the form
2.14
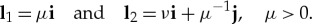
Here the constants *μ*, *ν* are unknown and form part of a solution to the hydroelastic problem.

*Isotropic case*. When the elastic sheet has zero density it may be convenient to consider stretching energy in the form

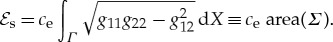
In this case, which is isotropic, the total elastic energy 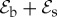 does not depend on a parametrization of *S* and is a geometric invariant. Consequently, it is not possible to determine the stretches in the elastic sheet in this case, see remark 3.1.

### (f) Regularization

Since general surfaces with finite Willmore energy need not be smooth, it is reasonable to consider possible regularizations of the energy functional which are compatible with the basic principles of mechanics. A natural way to do this is to adopt the so-called Cosserat theory of shells, see Antman [29], in which an elastic sheet is regarded as a two-dimensional medium with extrinsic directors. A special Cosserat shell is then a material surface *S* on which are defined several fields of vectors, called directors, and the Kirchhoff assumption is that there is the only director field, namely the field of unit normal vectors ***n***(*X*) to *S* at ***r***(*X*). (As before, *x* = ***r***(*X*), for *X* in the reference frame, is a parametrization of *S*.) For hyperelastic materials, the state of the elastic shell would then be completely characterized by an energy density of the form *E*_e_(∂_*α*_***r***, ∂_*β*_***n***). However, for general functions *E*_e_, the elastic energy density is not frame independent and would not be accepted as physically reasonable. However, the class of physically suitable stored energy functions includes [29] all functions *E*_e_ of the form *E*_e_ = *E*_e_(*g*_*αβ*_,  *b*_*αβ*_), where *g*_*αβ*_ and *b*_*αβ*_, the coefficients of the first and the second fundamental forms are given by (2.5) and (2.7). Thus, functionals
2.15
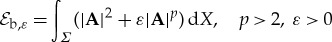
satisfies the frame independence principle and are physically admissible. Notice that every surface with bounded energy 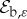 belongs to the class *C*^1+*α*^. So this class of functionals can be considered as a useful regularizations.

## 3. Variations, dynamic boundary conditions and stretches

Recall that the Lagrangian 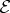 in (2.1) depends on the surface *S*, a parametrization ***r*** of *S* that satisfies (1.3), and parameters *ν*, *μ* which define the periods ***l***_*i*_ by (2.14). Here we calculate normal and intrinsic variations of 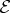 in order to derive the Euler equation and dynamic boundary conditions for the fluid, and to obtain the field of stretches, that characterize its critical points.

Let (*ν*_*t*_, *μ*_*t*_) be a *C*^1^ family of parameters with *μ*_*t*_ > 0, let ***r***_*t*_ be *C*^1^ family of smooth maps satisfying (1.3) where the periods ***l***_*i*,*t*_ are defined by (2.14), with (*ν*, *μ*) replaced by (*ν*_*t*_, *μ*_*t*_), and let *S*_*t*_ be the corresponding parametrized family of surfaces. Assuming that *S*_0_ = *S*, ***r***_0_ = ***r*** and (*ν*_0_, *μ*_0_) = (*ν*, *μ*), the variation of 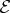 at (*S*, ***r***), (*ν*, *μ*) is defined as

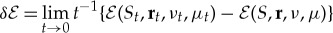
and (*S*, ***r***), (*ν*, *μ*) is a critical point of 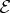 if

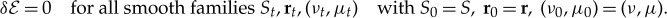
Note that the mappings ***r***_*t*_ uniquely define the surfaces *S*_*t*_, but ***r***_*t*_ and (*ν*_*t*_, *μ*_*t*_) can be considered as independent variables. Therefore, variations with respect to (*S*, ***r***) and to (*ν*, *μ*) can be calculated separately. For a fixed (*ν*, *μ*), we distinguish the normal and intrinsic (tangent) variations of ***r*** and *S*. To calculate normal variations, we use families of mappings of the form ***r***_*t*_(*X*) = ***r***(*X*) + *tψ*(*X*)***n***(*X*), where *ψ* is an arbitrary smooth, ***l***-periodic function. For intrinsic variations, we use families of mappings of the form ***r***_*t*_(*X*) = ***r***(*X* + *tϕ*(*X*)), where ***ϕ*** = (*ϕ*_1_, *ϕ*_2_) is an arbitrary smooth ***l***-periodic mapping.

### (a) Normal variations

The normal variations of the components of 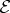 in (2.1) are
3.1
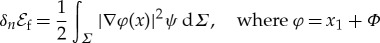
and
3.2
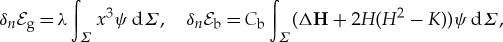
where Δ is the Laplace–Beltrami operator on *Σ* and, when the difference between the stretching energy and the inertial term is given by (2.12),
3.3
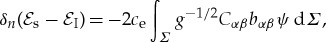
where the coefficients *b*_*αβ*_ of the second fundamental form in (2.7). Formulae (3.1) and (3.2) for 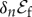 and 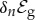 follow from well-known formulae for variations of Dirichlet-type integrals with respect to domain perturbations. Such results date back to Hilbert. Formula (3.2) for 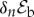 represents the famous Blaschke–Thomsen–Willmore expression [23] for variations of the Willmore energy. Relation (3.3) is obtained by straightforward calculations. If (*S*, ***r***), (*ν*, *μ*) is a critical point of the functional 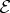, then relations (3.1)–(3.3) imply the dynamic condition on the free surface
3.4
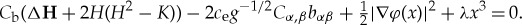


### (b) Intrinsic variations

The intrinsic variations of 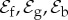 are zero because the renormalized kinetic energy and gravitational potential energy of the fluid, and the bending energy of the surface are geometric invariants that are independent of the parametrization. To calculate the intrinsic variation 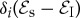 in (2.12), let

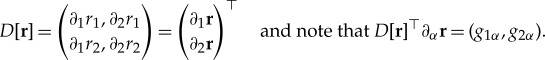
Since ***r***_*t*_(*x*) = ***r***(*x* + *t****ϕ***(*x*)), ***ϕ*** = (*ϕ*_1_, *ϕ*_2_), ∂_*α*_***r***_*t*_ = ∂_*α*_***r*** + *tD*[∂_*α*_***r***]***ϕ*** + *tD*[***r***]∂_*α*_***ϕ*** + *o*(*t*^2^) and so

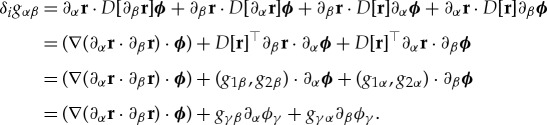
Hence by the divergence theorem and the symmetry of **C**,
3.5
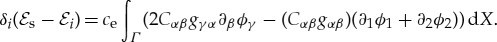
It follows that if (*S*, ***r***), (*ν*, *μ*) is a critical point of 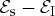, then the coefficients *g*_*αβ*_ of the first fundamental form satisfy the equations
3.6
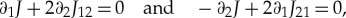
where, by the symmetry of the matrices ***C*** and ***g***,

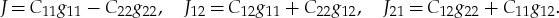
A further calculation yields that when the variations of 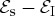 with respect (*ν*, *μ*) are zero,
3.7
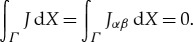
Hence, by (3.6), at criticality the ***l***-periodic function *J* satisfies the elliptic equation

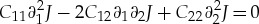
and hence, by (3.7), *J* = *J*_*αβ*_ = 0. It follows that at a critical point the first fundamental form in the reference frame has an ‘almost conformal’ structure

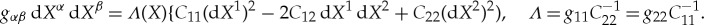
This can be regarded as the equation for the stretches in the elastic sheet. Note that the linear transform of the reference variables

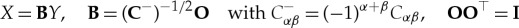
defines the isothermal coordinates on *S* with the conformal factor *e*^2*f*^ = *Λ*.

**Remark 3.1.**

In the isotropic case with 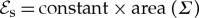 and with ϱ = 0, the intrinsic (tangent) variation of the Lagrangian 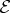 is identically equal to zero, and the normal variation of the stretching energy coincides with the mean curvature ***H***, up to a constant multiplier. In this particular case, the hydroelasticity equations do not determine the field of stretches in the elastic sheet.

## 4. Variational problem: existence of critical points

A variational problem for travelling hydroelastic waves can be formulated as follows. Fix spatial periods ***t***_*i*_, positive constants *C*_b_, *c*_e_, λ and a symmetric positive matrix ***C*** with 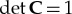. Let 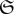 be an admissible set of parameters (*ν*, *μ*) and surfaces *S* which have continuous parametrizations ***r*** satisfying periodicity condition (1.3). The goal then would be to find non-trivial critical points (*S*, ***r***), (*ν*, *μ*) of the Lagrangian 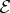 in 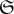. But this is not the only possible variational formulation. For example, it could be that some of the parameters *C*_b_, *c*_s_ or λ are unknown and should be regarded as parts of a solution to the problem. Also there may be additional constraints. One such constraint is immediately obvious. Since the equations are translationally invariant with respect to the space variables, it is necessary to prevent *S* from being unbounded in the vertical direction. To deal with this, we add the constraint (see definition 4.1 and lemma 4.2) that
4.1
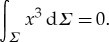


### (a) Properties of the Willmore energy and admissible sets

Admissible surfaces should satisfy minimal regularity conditions and critical surfaces should be physically realistic, in particular, they should have no self-intersections. Unfortunately, however, surfaces with square-integrable mean curvature may have singularities if the Willmore energy exceeds a certain critical value, see [24,25] for a discussion. For closed surfaces, this critical value is 8*π*. For the simple lattice with spatial periods ***t***_1_ = ***i*** = (1, 0, 0), ***t***_2_ = ***j*** = (0, 1, 0), define an admissible set as follows.

**Definition 4.1.**

For ***t***_1_ = ***i***, ***t***_2_ = ***j*** and positive constants E*, V*, denote by 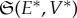 the set of all pairs (*S*, ***r***), (*ν*, *μ*) such that *S* admits a parametrization ***r*** satisfying (4.1) and the periodicity condition (1.3), where the periods ***l***_*i*_ are related to the parameters (*ν*, *μ*) by (2.14), and the periodic cell *Σ*⊂*S* has elastic energies bounded by constants E* and V* as follows:
4.2
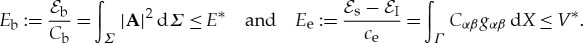

Surfaces of class 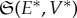 have bounded Willmore energy. In particular, they meet all requirements of the Hélein–Toro theorem [16], and hence admit a periodic isothermal bi-Lipschitz parametrization. The following lemmas record basic properties of elements of 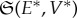.

**Lemma 4.2.**

*For an arbitrary element* (*S*, ***r***), (*ν*, *μ*) *of* 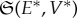 *there is a torus* 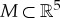 *and an immersion* *S* → *M* *such that*
4.3
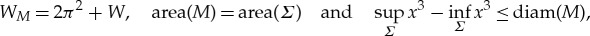
*where* *W*_*M*_ *and* *W* *are the Willmore energies of* *M* *and* *Σ*. *The torus* *M* *self-intersects if and only if the surface* *S* *self-intersects.*

**Lemma 4.3.**

*There is a constant* *c* > 0, *depending only on* V* *and* E*, *such that*
4.4
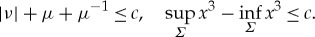

**Lemma 4.4.**

*Let* *δ*∈(0, 1) *and* E* = E*(*δ*): = 32*π* − 8*π*^2^ − *δ*. *Then the surface* *S* *has no self-intersections. Moreover*, *there exist a constant* *c* > 0 *and an exponent* *ι*∈(0, 1), *depending only on* V* *and* *δ*, *such that* ∥***r***∥_*C*^*ι*^(*Γ*)_ ≤ *c*.

*Sketch of proofs.* The existence of the torus *M* is by explicit construction and estimate (4.4) for *ν* and *μ* follows from (1.3), the second inequality in (4.2) and Bessel's inequality for trigonometric sums. Since, by [26, Lemma 1.1], diam *M* ≤ *c*(area *M*)^1/2^*W*^1/2^_*M*_ and since area *Σ* ≤ cV*, the second estimate in (4.4) follows by (4.3). The absence of self-intersections of *S* is based on a famous theorem of Li–Yau [30] by which the torus *M* has no self-intersections if *W*_*M*_ ≤ 8*π*. From this and lemma 4.1, we conclude that *S* has no self-intersections if *W* ≤ 8*π* − 2*π*^2^. It remains to note that *W* ≤ E*/4. Hölder estimates for ***r*** are proved by estimating logarithmic potentials in Orlicz spaces using an approach developed in [25]. Lemmas 4.3–4.4 imply the following compactness result for 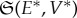.

**Lemma 4.5.**

*For* *δ*∈(0, 1) *and a sequence* {(*S*_*k*_, ***r***_*k*_), (*ν*_*k*_, *μ*_*k*_)} *in* 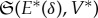 (*see lemma* 4.4) *there is a subsequence and* ((*S*, ***r***), 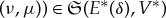 *such that* ***r***_*k*_ *converges uniformly to* ***r***, (*ν*_*k*_, *μ*_*k*_) *converge to* (*ν*, *μ*) *and*

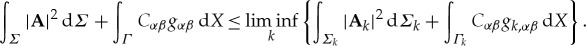

### (b) Existence of critical points: model problem

These results mean that the variational problem in the set 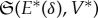 has at least one critical point when the parameters *C*_b_ and *c*_e_ are not specified *a priori* but are part of the solution. To see this, for fixed *δ*∈(0, 1) and V* > 0, introduce smooth, strictly monotone, convex functions 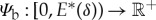, 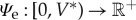 such that

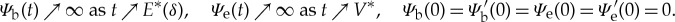
Now, for a modified Lagrangian 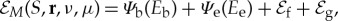 where *E*_b_ and *E*_e_ are defined in (4.2), consider the minimization problem

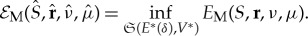
Because of lemma 4.5, there is at least one solution. The corresponding surface 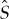 is not flat because the second variation of 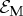 at the flat surface *S*_0_ = {*x*^3^ = 0} is the same as the second variation of the wave energy 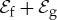 which, in turn, coincides with the second variation of the gravity wave energy which equals the quadratic form corresponding to the equations of linear gravity wave theory. However, the latter is indefinite since the corresponding dispersion function *k*^2^_1_ − λ|***k***| changes sign at an infinite set of wavevectors ***k***. In other words, a flat solution is a saddle point for 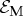. (Note that this holds only in three dimensions.) It is easy to check that a minimizer of 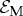 is a critical point of the Lagrangian 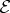 and its regularity can be established by using the hole-filling and bi-harmonic approximation methods proposed in [26]. The detailed discussion of these issues is beyond the scope of this paper.

## 5. Conclusion

The main outcome is confirmation that certain mathematical aspects of the existence and properties of hydroelastic waves have much in common with the modern geometric theory of surfaces; in particular, those that are critical with respect to the Willmore functional. The details, which are extensive, will appear elsewhere.

## Data accessibility

This article has no additional data.
